# Effects of water access time and unlimited access to alfalfa straw on litter quality, performance, and behavior of breeder pullets

**DOI:** 10.1016/j.psj.2024.103773

**Published:** 2024-04-16

**Authors:** R.A. van Emous

**Affiliations:** Wageningen Livestock Research, Wageningen, the Netherlands

**Keywords:** broiler breeder pullet, water access time, alfalfa straw, litter quality, performance

## Abstract

Between 3 and 20 wk of age (**WOA**), the effects of water access time and access to alfalfa during the rearing phase on the litter conditions, performance, and behavior of broiler breeder pullets was studied. A total of 480 female one-day-old chicks (Ross 308) were randomly assigned to 24 floor pens (20 pullets/pen) within a 3 × 2 factorial completely randomized block design. Between 3 and 20 WOA, pullets received water 1) between 07:30 am and 10:30 pm h (**3HR**), 2) in 2 periods between 07:30 am and 11:00 pm h and between 14:00 pm and 15:30 pm h (**5HR**), or 3) during the entire light period (**8HR**). Half of the pens had unlimited access to alfalfa straw (**ALF**+) or not (**ALF**-). Higher water use and water-to-feed ratios were observed in the 5HR and 8HR pullets compared to the 3HR pullets (*P* < 0.001), with no effect observed from unlimited alfalfa. Clear differences in water use throughout the day were observed for the different water strategies. The dry matter (**DM**) content in the litter was lower, and the litter friability and moisture scores were higher in the 5HR and 8HR than the 3HR pens (*P* < 0.001), with no differences in fresh feces DM. Alfalfa straw had no effect on litter DM content, fresh feces DM content, litter friability score, or litter moisture score. Feather cover score and feather and footpad contamination score were higher in 5HR and 8HR pullets than in 3HR pullets (*P* < 0.05), with no differences between the ALF+ and ALF- pullets. The 5HR and 8HR pullets showed increased pecking at alfalfa straw and drinking nipples, along with decreased foraging and perching than the 3HR pullets (*P* < 0.05). Additionally, ALF+ pullets showed a tendency for less object pecking behavior (*P* = 0.066) than ALF- pullets. In conclusion, the study demonstrated that extended access to water in breeder pullets increased water use, resulting in inferior litter quality, decreased feather cover, and decreased feather cover and footpad contamination. Moreover, unlimited access to alfalfa straw decreased object pecking behavior.

## INTRODUCTION

Unrestricted access to water and alfalfa straw appear to be crucial factors for improving the behavior and welfare of breeders. Broiler breeders, particularly during the rearing phase, are subjected to controlled feed intake to regulate their growth rate. To prevent wet litter conditions, a restricted amount of water is also provided. Under Nortwest Europe commercial circumstances, water is typically provided for 3 h daily during the rearing phase and for 5 to 7 h daily during the laying phase (van Emous, 2022). Approximately 65% of rearing and breeder farmers apply a maximum time limit on water access, while other farmers apply a maximum amount of water based on a water-to-feed ratio of around 1.8. Notably, the literature on water restriction is limited, with only 2 dated articles from the same experiment available (Hocking, 1993; Hocking et al., 1993). The study by Hocking et al. (1993) showed that water restriction with water available 2 to 3 h per day had no effect on the measured welfare indicators, such as plasma corticosterone concentration, heterophil/lymphocyte ratio in the blood, plasma viscosity, or total oral stereotypic pecking behavior, when compared to unlimited water provision. Additionally, Hocking (1993) concluded that pullets with limited morning access to water (2–3 h) did not show noticeable signs of thirst. In the experiment of Hocking et al. (1993), water restriction resulted in drier litter compared to unlimited water access, leading to the conclusion that constant water access may not be necessary for rearing pullets.

Recently, there has been a growing body of evidence derived from experiments involving broiler breeders provided with unrestricted water access throughout both the rearing and laying phases. In our laboratory experiments, a significant disparity in water-intake behavior among adult breeders became apparent when subjected to different water management practices during the rearing phase. Specifically, breeders with 7.5 h access to water within the 8-h photoperiod during rearing had lower water intake at the onset of the laying period compared to pullets with restricted water access during rearing (van Emous, 2022). Notably, pullets that received unrestricted water throughout the entire photoperiod during rearing displayed an elevated water-to-feed ratio (2.3 vs. 1.8), but this increase did not result in detrimental effects on litter quality.

Over the last 50 yr, there has been a remarkable increase in the growth potential of broiler chickens and consequently broiler breeders (Zuidhof et al., 2014). For this reason, the amount of feed that broiler breeder pullets receive during rearing is controlled. Two to 3 decades ago, literature assumed a feed control level during the rearing phase ranging from 66 to 75% compared to ad libitum feed intake (de Jong et al., 2002). More recently, Carney et al. (2022) compared 4 different broiler breeder strains, fed either ad libitum or restricted. These included 2 unselected strains dating back to 1957 and 1978, as well as 2 randomly bred strains originating from 1995 and 2025. To maintain the different restricted-fed pullets on target BW, feed restriction level was 1, 43, 70, and 75% for the 1957, 1978, 1995, and 2015 strains, respectively. Due to the ongoing genetic development of broiler BW growth, it can be hypothesized that feed restriction level for modern breeders is even higher.

Behavioral research has consistently indicated that feed restriction in broiler breeder pullets leads to abnormal behavior, indicating hunger and frustration. This manifests in stereotypical pecking behavior toward objects, such as walls, empty feeding systems, and empty water systems (Hocking et al., 1996, 2001; Savory and Kostal, 1996; de Jong et al., 2002). Alfalfa straw is a common variant of roughage with low energy and high fiber content, and alfalfa is assumed to help reducing stereotypical behavior (Elmutalab, 1998; Verwer and Wagenaar, 2009; Hu et al., 2021).

It is believed that the prolonged presence of fibers in the gizzard induces a sense of satiety in chickens (van Krimpen et al., 2008). De Jong et al. (2005) explored the effects of increased fiber levels and different fiber types during the rearing and laying phases. They found that a diet containing added fiber (21% diluted diet) had positive effects on behavior in the first half of the rearing phase. Moreover, studies on laying hens demonstrated that the unlimited provision of alfalfa as an enrichment material contributed to the prevention of feather pecking, resulting in improved feather cover (Schreiter et al., 2020; Tainika and Şekeroğlu, 2021). Currently, alfalfa straw is provided to mini slow-growing breeders, but there is limited experience with providing alfalfa straw to regular broiler breeders. This experiment, therefore, aimed to investigate the effects of different water access time and the provision of unlimited access to alfalfa straw on litter quality, performance, and behavior in broiler breeder pullets.

## MATERIALS AND METHODS

### Experimental Design

**Figure 1:**
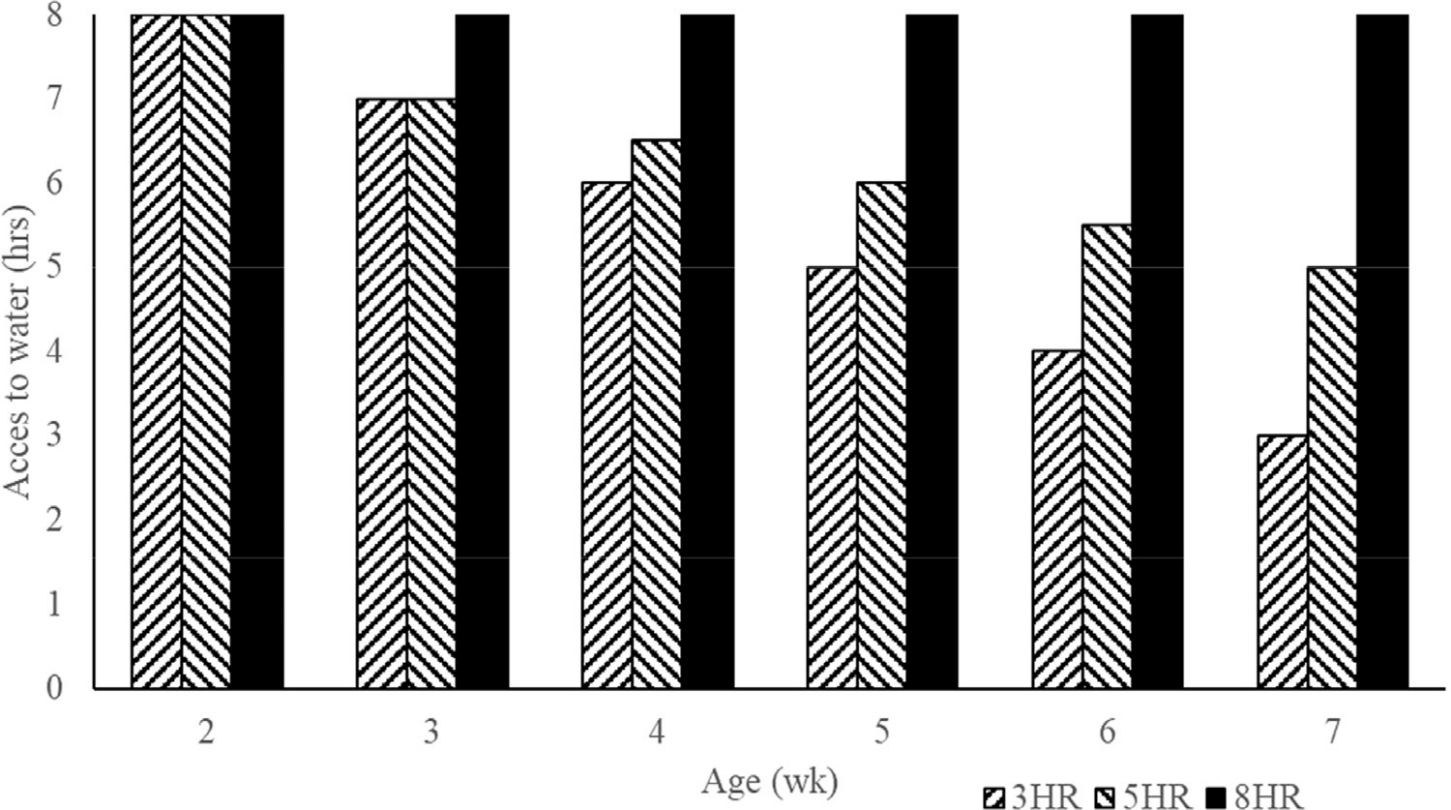
Development of water access period (hours) per treatment in the intermediate period (between 2 and 7 WOA).

The study was performed using a 3 × 2 factorial completely randomized block (room) design incorporating 3 different water access time and either unlimited access or no access to alfalfa straw. The experiment timeline comprised a pre-period (0–3 WOA), an intermediate period (3–7 WOA), and an experimental period (7–20 WOA). During the preliminary period, all pullets received 8 h water. In the intermediate period, access to water for pullets in the 3HR and 5HR groups was gradually reduced to the treatments final duration of water access (Figure 1). This was done because an abrupt transition from 8 h to 3 or 5 h access to water was not in accordance with commercial circumstances. In the experimental period, pullets had access to water during different time frames: 1) between 07:30 am and 10:30 pm h (**3HR**), 2) in 2 periods between 07:30 am and 11:00 pm h and between 14:00 pm and 15:30 pm h (**5HR**), or 3) throughout the entire light period (**8HR**). Half the pullets had unlimited access to alfalfa straw (**ALF+**), while the other half did not (**ALF-**). Alfalfa straw was provided in one small hanging plastic bucket with 4 holes per pen. Alfalfa contains approx. 91% DM, 17% CP, 2.5% CF, and 31.5% CF (Timmerman Luzer, Kortgene, the Netherlands).

### Housing and Management Breeder Pullets

A flock of 480 Ross 308 female broiler breeder 1-day-old chicks, obtained from Aviagen-EPI, Roermond, the Netherlands, were distributed across 24 floor pens measuring 2.5 × 1.0 m each, in 2 identical climate-controlled rooms. The study started at d 21, with 20 pullets per pen. An additional pen with 40 pullets was reserved to replace dead or culled pullets between d 1 and 21. The pen floors contained wood shavings as bedding material (2.0 kg/m^2^). Initially, feed was provided using 3 manual round feeders up to 3 WOA. From 3 WOA onward, feed was provided in 1 feeding trough (2.0 m length) and, from 10 WOA, in 2 feeding troughs (2.8 m). Water was administered via a water system with 5 nipple drinkers with drip cups positioned above the litter floor. Between 0 and 10 WOA pullets had access to 2 wooden perches at different heights (2 m length in total), and from 10 WOA onward to 2 wooden perches and one plastic perch (3 m length in total).

Throughout the experiment, all birds across different treatments were maintained at the same target BW. Feed allocation was adjusted to adhere to the predetermined body growth curve following the guidelines provided by the breeder company (Aviagen, 2021). Feed was provided ad libitum (at 07:45 am) from 0 to 2 WOA, and from 2 WOA pullets were fed a controlled amount of feed. The pullets followed a standard 3-phase rearing feeding program with “Starter-1″ from 0 to 3 WOA, “Starter-2″ from 3 to 10 WOA, and “Grower” from 10 to 20 WOA (Table 1). Additionally, feed pellets (2 grams per pullet) were distributed onto the litter every afternoon as scratch feed.

**Table 1:** Dietary ingredients and calculated nutrients of the pullet diets (g/kg, as-fed basis).

Item	Starter-1^1^ (0 to 3 WOA)	Starter-2^2^ (3 to 10 WOA)	Grower^3^ (10 to 20 WOA)
Ingredient			
Maize	353.7	340.0	327.8
Wheat	178.6	295.5	246.4
Oats	15.0	20.0	25.0
Oat hulls	5.0	-	10.0
Maize gluten feed 60%	5.0	-	5.7
Maize solubles	-	-	5.0
Wheat gluten feed	-	-	19.3
Wheat middlings	40.0	42.7	100.0
Maize DDGS	-	15.0	-
Rapeseed	-	30.0	50.0
Soybean meal	234.1	134.7	16.2
Sunflower meal LP	-	-	50.0
Sunflower meal MP	-	50.0	100.0
Sunflower meal HP	47.5	23.7	-
Peas	15.0	10.0	-
Vinasse	10.0	-	10.0
Palm kernels	-	5.0	-
Palm oil	5.0	-	-
Soy oil	5.0	-	-
Salm oil	5.0	3.0	3.0
Lecithin mix	11.3	4.8	-
Acid mix	1.5	4.3	4.7
Limestone (course)	-	-	8.7
Chalk	13.8	15.7	3.0
Monocalcium phosphate	7.9	6.8	2.3
Sodium-Bicarbonate	3.6	2.8	2.3
Salt	13.8	15.7	3.0
DL-Methionine	3.3	2.4	1.3
L-Lysine	1.4	0.2	0.9
L-Threonine	2.1	1.3	1.2
L-Tryptophane	-	0.1	-
L-Valine	-	0.2	-
Mineral-Enzyme mix	5.4	5.7	1.8
Premix	4.0	4.0	4.0
Calculated content			
AME_n_ (kcal/kg)	2,785	2,700	2,460
Crude ash	59.3	57.4	49.6
Crude protein	195.0	170.1	148.3
Crude fat	50.0	36.3	28.0
Crude fiber	37.5	45.7	71.1
Starch	385.1	418.7	408.5
C18:2 linolenic acid	20.5	14.8	13.1
Calcium	8.86	9.61	7.17
Absorbable phosphorus	4.29	4.20	3.30
Sodium	1.83	1.80	1.60
Potassium	8.65	7.63	7.30
Chloride	2.00	2.00	1.95
DEB (mEq/kg)	94.6	85.3	78.6
SID Lysine	9.52	6.75	5.20
SID Met+Cys	8.82	7.55	6.37
SID Threonine	7.98	6.34	5.36
SID Tryptophane	1.96	1.75	1.45
Soluble NSP	30.8	31.4	32.8

Abbreviation: WOA, weeks of age.

1 Provided per kilogram of complete diet: vitamin A, 10,000 IU; vitamin B1, 4.8 mg; vitamin B2, 6.0 mg; vitamin B3, 52.0 mg; vitamin B4, 369.5 mg; vitamin B5, 22.3 mg; vitamin B6, 5.8 mg; vitamin B9/B11, 2.6 mg; vitamin B12, 0.04 mg; vitamin D3 + D3OH, 3,000 IU; vitamin E, 65.0 mg; vitamin H, 0.38 mg; vitamin K3, 3.9 mg; iron, 261.8 mg; copper, 21.9 mg; manganese, 129.5 mg; total zinc, 106.3 mg; iodine, 1.7 mg; selenium, 0.58 mg.

2 Provided per kilogram of complete diet: vitamin A, 10,000 IU; vitamin B1, 4.2 mg; vitamin B2, 6.0 mg; vitamin B3, 54.0 mg; vitamin B4, 342.9 mg; vitamin B5, 20.3 mg; vitamin B6, 5.2 mg; vitamin B9/B11, 2.6 mg; vitamin B12, 0.04 mg; vitamin D3 + D3OH, 3,000 IU; vitamin E, 68.5 mg; vitamin H, 0.38 mg; vitamin K3, 3.6 mg; iron, 275.1 mg; copper, 21.8 mg; manganese, 125.2 mg; total zinc, 113.8 mg; iodine, 2.3 mg; selenium, 0.58 mg.

3 Provided per kilogram of complete diet: vitamin A, 10,000 IU; vitamin B1, 3.0 mg; vitamin B2, 9.0 mg; vitamin B3, 32.5 mg; vitamin B4, 400.0 mg; vitamin B5, 16.3 mg; vitamin B6, 4.0 mg; vitamin B9/B11, 2.0 mg; vitamin B12, 0.02 mg; vitamin D3 + D3OH, 3,000 IU; vitamin E, 100 mg; vitamin H, 0.028 mg; vitamin K3, 3.0 mg; iron, 180.4 mg; copper, 23.4 mg; manganese, 131.5 mg; total zinc, 115.6 mg; iodine, 2.0 mg; selenium, 0.50 mg.

Room temperature was maintained at 35°C during the first 2 d, and from d 3 onwards temperature was gradually reduced to 20°C by wk 4. The pullets were subjected to a photoperiod of 24L:0D (40 lx) for the first 3 d, which was gradually reduced to a photoperiod of 8L:16D (5 lx) by 3 WOA, with lights on between 07:30 am and 15:30 pm h. Pullets were non-beak-trimmed and vaccinated according to a standard commercial protocol (Aviagen-EPI, Roermond, the Netherlands).

The protocol for the experiment was approved by the Dutch Central Committee on Animal Testing, Den Haag, the Netherlands (approval number: AVD4010020185007) and the Institutional Animal Care and Use Committee.

### Observations

#### Body Weight and BW Uniformity

To monitor BW and BW gain, all pullets were weighed as group on a weekly basis from 0 to 20 WOA in the morning prior to feeding. BW uniformity was determined at 5, 10, 15, and 20 WOA based on the coefficient of variation (**CV**) and calculated by dividing the SD per pen by the average body weight per pen.

#### Water Use, Feed Intake, and Water-to-Feed Ratio

Weekly water usage was determined by reading the water level on the water container (25 mL scale) for each pen, and water and feed intake was calculated for both the rearing and laying phases. The water-to-feed ratio was calculated by dividing the cumulative water intake by the cumulative feed intake for each phase.

#### Water Use During the Day

Water use throughout the day was determined by reading the water level on the water container (25 mL scale) at specific intervals at 7, 10, 12, 15, 17, and 20 WOA at 4 different times (09:30 am, 11:30 am, 13:30 pm , and 15:30 pm h).

#### Alfalfa Straw Use

The alfalfa straw in the buckets was supplemented on Fridays and Mondays to 300 g between 3 and 10 WOA, and to 500 g from 10 WOA onward, ensuring that the pullets had constant access to sufficient supply of alfalfa straw. The remaining amount of alfalfa straw was weighed back on Fridays and Mondays. Alfalfa straw use was calculated by subtracting the amount of alfalfa at the end from the amount of alfalfa at the beginning.

#### Litter and Fresh Feces Characteristics

At 7, 10, 13, 15, 17, and 20 WOA, DM content of the litter was determined by collecting a 500 g representative sample directly below the nipple-drinkers system. The sample was specifically taken in this location to provide insights into water spillage. After mixing, a subsample of 200 g was extracted for DM content analysis. Fresh feces were collected at 7, 10, 13, 15, 17, and 20 WOA to determine DM content. To collect fresh feces, a low container (5 cm height) with dimensions of 50 cm in depth and 30 cm in width, equipped with a mesh lid, was positioned in the pens directly after lights were turned on. Subsequently, fresh feces were collected from the containers every 2 h and a 200 g subsample was extracted for DM content analysis. The DM analysis involved gravimetric determination following oven drying for 4 h at 103°C (±3°C) in accordance with the NEN 7432 standard (1996).

Litter quality was visually scored at 10, 15, and 20 WOA. The friability of the litter in each pen was scored on a scale ranging from 0 (no cake) to 5 (100% cake). The wetness of the litter in each pen was scored on a scale ranging from 0 (very dry) to 4 (very wet).

#### Feather Cover Quality and Contamination of Feather Cover and Foot Pads

At 10, 15, and 20 WOA, the feather cover quality of 5 pullets per pen was evaluated using the Bilcik and Keeling method (1999). Pullets were randomly caught by the staff and placed in crates in front of the compartment. Scoring was performed on 7 parts of the body (neck, breast, belly, back, wings, tail, and thighs), with scores ranging from 0 (totally intact surface) to 5 (totally bald surface). Additionally, the contamination of feather cover and foot pads was scored on a scale of 0 to 3: 0 (clean), 1 (somewhat dirty), 2 (dirty), and 3 (filthy).

#### Behavioral Observations

Behavioral observations were conducted by 2 persons at 10, 15, 20 WOA, following an ethogram developed by van Emous et al. (2015). This was performed by using the scan sampling method, and pullet behavior was recorded 8 times (07:30 am, 08:30 am, 09:30 am, 10:30 am, 11:30 am, 12:30 pm, 13:30 pm, and 14:30 pm h) per observation day per pen. The number of pullets showing various behaviors, such as eating, pecking at alfalfa, drinking, standing, sitting, walking, foraging, comfort, dust-bathing, object-pecking, aggressive pecking, sitting on perch, and standing on perch, was recorded at each observation session. Object pecking included pecking at parts of the pen, wall, empty feeders, empty drinkers, and to the alfalfa bucket.

### Statistical Analysis

The data were analyzed using Genstat statistical software (Genstat, 2022) and pen was the experimental unit and results are shown as nontransformed means with corresponding standard errors. Differences between means were reported as significant where *P* ≤ 0.05 and trend were reported where 0.05 ≤ *P* < 0.10. Response variables regarding water-use, water-to-feed ration, and alfalfa use were analyzed using the ANOVA (Analysis of Variance) procedure of GenStat according to the following model:

$$Y_{\text{ijk}}=\mu +R_{i}+W_{j}+L_{k}+W_{j}*L_{k}+\varepsilon_{\text{ijk}}$$

where Y_ijk_ is the response variable, μ the overall mean, R_i_ the random effect of room (i = 1, 2), W_j_ the effect of water access time (3HR, 5HR, and 8HR; j = 1, 2, 3), L_k_ the effect of whether or not access to alfalfa was given (ALF+, ALF-; k = 1, 2), and Ɛ_ijkm_ the residual error term. Room and pen within room were included in the model as random terms.

Response variables regarding feather cover score, contamination of feather cover score, foot pads score, and behavior were analyzed using the Generalized Linear Mixed Model (**GLMM**) procedure using logistic regression and Poisson distribution of GenStat.

The statistical model for BW uniformity, DM litter, DM fresh feces, litter-friability score, litter-wetness score, feather cover quality, feather cover and footpad contamination, and behavior included age as a fixed effect.

## RESULTS

No meaningful significant interactions were observed between water access time and unlimited access to alfalfa straw. Consequently, only the main effects of the treatments will be discussed.

### Body Weight and BW Uniformity

As a result of following the same BW target curve, no differences appeared in BW at any age (data not shown). Throughout the entire experimental period, the BW of the pullets generally exceeded the BW recommendations of the breeding company with on average 115 g (Aviagen, 2021). There were no differences in BW uniformity among pullets subjected to different water access times (Table 2). Pullets with unlimited access to alfalfa straw, however, showed a higher CV than pullets without access to alfalfa straw (11.8 vs. 10.4; *P* = 0.042). Additionally, BW uniformity worsened while aging (*P* < 0.001).

**Table 2:** Effects of water access time, unlimited access to alfalfa straw, and age on BW CV.

Treatment^1^	BC CV
Water access time	
3HR	11.3
5HR	11.1
8HR	11.0
SEM	0.57
Access to alfalfa	
ALF+	11.8^a^
ALF-	10.4^b^
SEM	0.46
Age	
5 WOA	10.0^c^
10 WOA	10.8^b^
15 WOA	11.8^a^
20 WOA	11.8^a^
SEM	0.23
*P*-value	
Water access time	0.909
Access to alfalfa	0.042
Age	< 0.001
Water × alfalfa	0.849
Water × age	0.516
Alfalfa × age	0.773
Water × alfalfa × age	0.021

Abbreviation: WOA, weeks of age.

a–c Means within a column with no common superscript differences (*P* ≤ 0.05).

1 3HR = 3 h water access between 07:30 am and 10:30 am h, 5HR = 5 h water access in 2 periods between 07:30 am and 11:00 am h and between 14:00 pm and 15:30 pm h, 8HR = 8 h water access during the entire light period. ALF+ = unlimited access to alfalfa straw, ALF- = no access to alfalfa straw. WOA = week of age.

### Feed Allowance and Alfalfa Straw Use

No differences were found for the different water access times during the entire experimental period and no differences between unrestricted access to alfalfa till 18 WOA (Table 3). Feed allowance was higher for the ALF- pullets in wk 19 and 20 compared to the ALF+ pullets. Alfalfa use in both the intermediate and experimental periods was higher in the 5HR and 8HR pullets than the 3HR pullets (Table 4).

**Table 3:** Effects of water access time, unlimited access to alfalfa straw, and age on feed allowance.

	Water access time			Access to alfalfa	
Age (wk)	3HR	5HR	8HR	ALF+	ALF-
4	36.0	36.0	36.0	36.0	36.0
5	41.0	41.0	41.0	41.0	41.0
6	45.0	45.0	45.0	45.0	45.0
7	53.0	53.4	53.1	53.2	53.2
8	55.8	56.4	56.0	56.0	56.1
9	57.0	57.1	56.9	56.8	57.2
10	58.6	58.6	58.8	58.6	58.7
11	63.0	63.0	63.0	63.0	63.0
12	65.0	65.1	65.0	64.9	65.2
13	69.0	69.0	69.0	69.0	69.0
14	72.9	73.3	73.1	72.8	73.3
15	74.9	74.8	75.3	74.8	75.2
16	76.0	76.0	76.0	76.0	76.0
17	76.9	76.6	77.1	76.8	77.0
18	80.0	79.9	80.1	79.8	80.3
19	81.9	81.9	82.0	81.4^b^	82.4^a^
20	85.1	85.1	85.5	84.9^b^	85.6^a^

a–b Means within a column and within main treatments with no common superscript differences (*P* ≤ 0.05). ^1^3HR = 3 h water access between 07:30 am and 10:30 am h, 5HR = 5 h water access in 2 periods between 07:30 am and 11:00 am h and between 14:00 pm and 15:30 pm h, 8HR = 8 h water access during the entire light period. ALF+ = unlimited access to alfalfa straw, ALF- = no access to alfalfa straw.

**Table 4:** Effects of water access time on alfalfa use (g/b/d) in the intermediate and experimental periods.

Treatment^1^	Intermediate period (3–7 WOA)	Experimental period (7–20 WOA)
3HR	1.2^b^	2.0^b^
5HR	2.0^a^	4.6^a^
8HR	1.8^a^	4.9^a^
SEM	0.13	0.59
*P*-value	0.010	0.016

a–b Means within a column with no common superscript differences (*P* ≤ 0.05).

1 3HR = 3 h water access between 07:30 am and 10:30 am h, 5HR = 5 h water access in 2 periods between 07:30 am and 11:00 am h and between 14:00 pm and 15:30 pm h, 8HR = 8 h water access during the entire light period. ALF+ = unlimited access to alfalfa straw, ALF- = no access to alfalfa straw. WOA = week of age.

### Water Use and Water-to-Feed Ratio

During the intermediate period (3–7 WOA), the 5HR pullets showed higher water use than the 3HR pullets (141.1 vs. 121.6 mL/b/d; *P* = 0.027), while the water use of the 8HR pullets did not differ from that of the 3HR or 5HR pullets (Table 5). In the experimental period (7–20 WOA), water use was higher in the 5HR and 8HR pullets than in the 3HR pullets (189.2 and 192.4 vs. 137.8 mL/b/d; *P* < 0.001).

**Table 5:** Effects of water access time, and unlimited access to alfalfa straw on water use and water-to-feed ration in the intermediate and experimental period.

	Intermediate period (3–7 WOA)		Experimental period (7–20 WOA)	
Treatment 1	Water use (mL/b/d)	Water-to-feed ratio	Water usage (mL/b/d)	Water-to-feed ratio
Water access time				
3HR	121.6^b^	2.84^b^	137.8^b^	1.96^b^
5HR	141.1^a^	3.29^a^	189.2^a^	2.68^a^
8HR	127.4^ab^	2.96^b^	192.4^a^	2.71^a^
SEM	4.71	0.109	6.59	0.100
Access to alfalfa				
ALF+	129.2	3.01	175.4	2.49
ALF-	130.9	3.05	170.8	2.41
SEM	3.85	0.089	5.38	0.081
*P*-value				
Water access time	0.027	0.025	< 0.001	< 0.001
Access to alfalfa	0.770	0.785	0.550	0.509
Water × alfalfa	0.124	0.122	0.806	0.823

a–b Means within a column with no common superscript differences (*P* ≤ 0.05).

1 3HR = 3 h water access between 07:30 am and 10:30 am h, 5HR = 5 h water access in 2 periods between 07:30 am and 11:00 am h and between 14:00 pm and 15:30 pm h, 8HR = 8 h water access during the entire light period. ALF+ = unlimited access to alfalfa straw, ALF- = no access to alfalfa straw. WOA = week of age.

The water-to-feed ratio in the intermediate period was higher in the 5HR pullets than the 3HR and 8HR (3.29 vs. 2.84 and 2.96 mL/d/d; *P* = 0.025). In the experimental period, the water-to-feed ratio was higher in the 5HR and 8HR pullets than the 3HR pullets (2.68 and 2.71 vs. 1.96 mL/d/d; *P* < 0.001). Providing unlimited access to alfalfa straw had no effect on water use or water-to-feed ratio.

### Water Use During the Day

**Figure 2:**
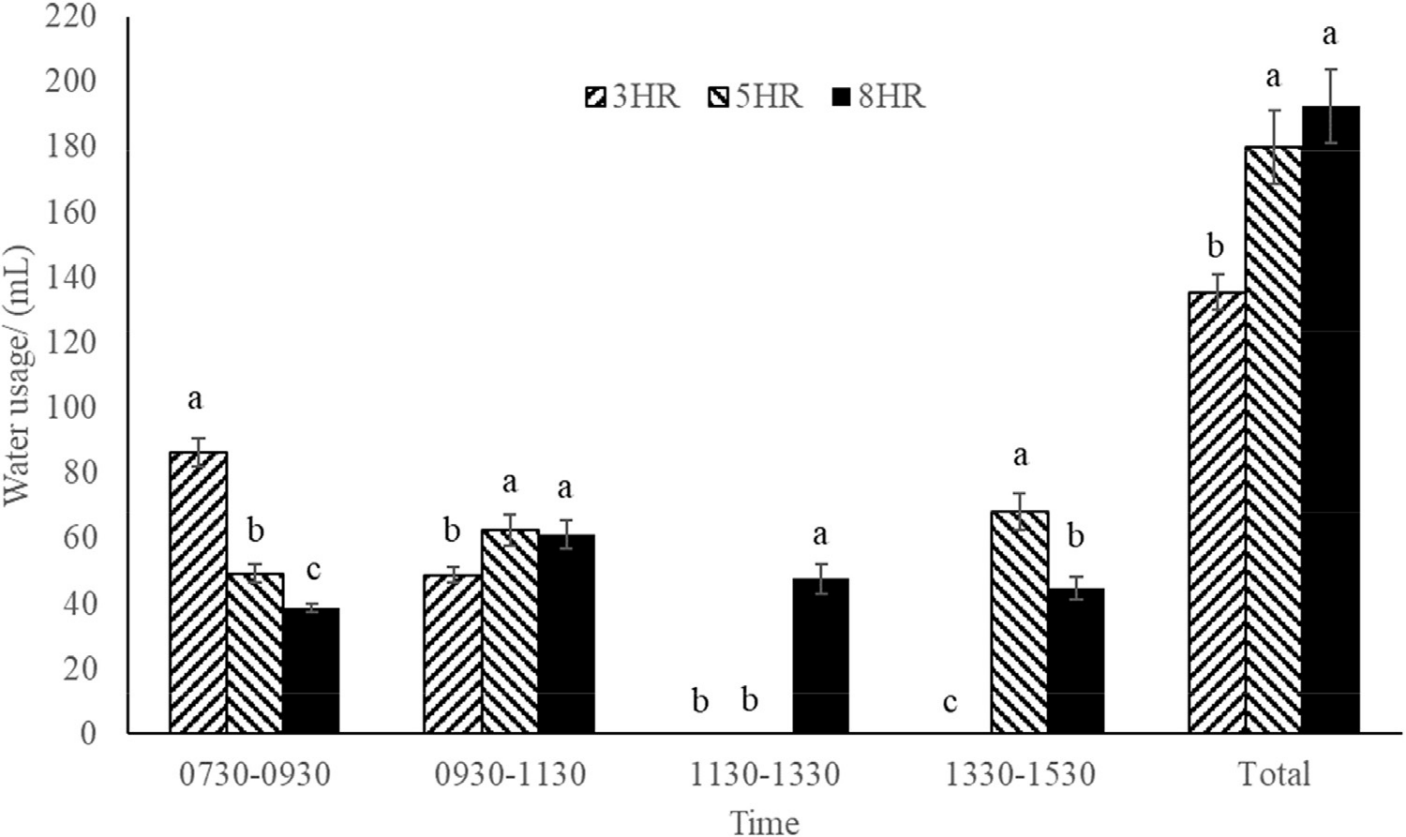
Water use (mL) throughout the day.

Clear differences in water use throughout the day were evident among the different water access time (Figure 2). Specifically, the 3HR pullets had water use between 07:30 am and 09:30 am h approximately twice as high as the 5HR and 8HR pullets. The 5HR pullets demonstrated lower water use in the morning between 07:30 am and 09:30 am h but slightly higher water use between 09:30 am and 11:30 am h and in the afternoon between 13:30 pm and 15:30 pm h. Conversely, the 8HR pullets showed a more consistent water-use pattern throughout the day, with a peak occurring between 09:30 am and 11:30 am h.

### Litter Characteristics

The average dry-matter content of the litter (below the drinking-nipple line) was lower (*P* < 0.001) in the 5HR (40.3%) and 8HR (43.9%) pens than the 3HR pens (66.0%; Table 6). There were, however, no differences between the different water access time in the dry-matter content of the fresh feces. The litter friability score was higher (4.7 and 4.4 vs. 2.6; *P* < 0.001) in the 5HR and 8HR pens than the 3HR pens. This indicates that the litter in the 5HR and 8HR pens was less loose and contained a higher proportion of caked litter surface. Additionally, the litter moisture score was also higher (4.4 and 3.9 vs. 1.9; *P* < 0.001) in the 5HR and 8HR pens than the 3HR pens, meaning that the litter in these pens was wetter.

**Table 6:** Effects of water access time, unlimited access to alfalfa straw, and age on DM litter, DM fresh feces, friability score and wetness score.

Treatment^1^	DM litter (%)	DM fresh feces (%)	Friability score^2^	Wetness score^3^
Water access time				
3HR	66.0^a^	22.6	2.6^b^	1.9^b^
5HR	40.3^b^	22.1	4.7^a^	4.4^a^
8HR	43.9^b^	22.7	4.4^a^	3.9^a^
SEM	3.00	3.18	0.21	0.24
Access to alfalfa				
ALF+	48.7	22.2	3.9	3.5
ALF-	51.5	22.7	3.9	3.3
SEM	2.44	2.60	0.17	0.20
Age				
7 WOA	52.9^b^	21.5^c^	3.5^c^	3.1^b^^c^
10 WOA	57.4^a^	21.9^c^	3.2^d^	2.5^d^
13 WOA	47.8^cd^	21.6^c^	4.0^b^	3.6^b^
15 WOA	45.6^d^	22.2^bc^	4.2^ab^	3.7^ab^
17 WOA	45.7^d^	23.3^ab^	4.3^a^	4.0^a^
20 WOA	51.0^bc^	24.3^a^	4.3^a^	3.5^bc^
SEM	1.42	4.82	0.11	0.13
*P*-value				
Water access time	< 0.001	0.386	< 0.001	< 0.001
Access to alfalfa	0.429	0.219	0.934	0.657
Age	< 0.001	< 0.001	< 0.001	< 0.001
Water × alfalfa	0.743	0.934	0.312	0.641
Water × age	0.003	0.143	0.196	0.020
Alfalfa × age	0.537	0.748	0.941	0.424
Water × alfalfa × age	0.998	0.993	0.813	0.989

Abbreviation: WOA, weeks of age.

a–d Means within a column with no common superscript differences (*P* ≤ 0.05).

1 3HR = 3 h water access between 07:30 am and 10:30 am h, 5HR = 5 h water access in 2 periods between 07:30 am and 11:00 am h and between 14:00 pm and 15:30 am h, 8HR = 8 h water access during the entire light period. ALF+ = unlimited access to alfalfa straw, ALF- = no access to alfalfa straw. WOA = week of age.

2 Friability of the litter was scored on a scale ranging from 0 (no cake) to 5 (100% cake).

3 Wetness of the litter was scored on a scale ranging from 0 (very dry) to 4 (very wet).

Providing alfalfa straw showed no effect on the dry-matter content of litter, dry-matter content of fresh feces, litter friability score, or litter moisture score.

The dry-matter content of the litter decreased between 7 and 17 WOA but was higher again at 20 WOA. By contrast, the dry-matter content of fresh feces increased between 7 and 20 WOA. The litter-friability and moisture scores increased (indicating a deterioration) with the aging of the pullets.

### Feather Cover Quality and Contamination of Feather Cover and Footpad

The average feather cover score for the 5HR and 8HR pullets was higher (indicating worse feather cover) than that of the 3HR pullets (0.71 and 0.75 vs. 0.45; *P* = 0.025; Table 7). These differences were caused primarily by tendencies to a higher score on the belly (1.52 and 1.43 vs. 0.29; *P* = 0.093) and on the tail (1.16 and 1.17 vs. 0.86; *P* = 0.064) for the 5HR and 8HR pullets.

**Table 7:** Effects of water access time, unlimited access to alfalfa straw, and age on feather cover score.^1^

Treatment^2^	Neck	Breast	Belly	Back	Wings	Tail	Thighs	Average
Water access time								
3HR	0.74	0.00	0.29	0.06	1.07	0.86	0.15	0.45^b^
5HR	0.70	0.10	1.52	0.03	1.15	1.16	0.33	0.71^a^
8HR	0.75	0.20	1.43	0.05	1.19	1.17	0.47	0.75^a^
SEM	0.058	0.079	0.298	0.022	0.072	0.103	0.136	0.083
Access to alfalfa								
ALF+	0.72	0.10	0.98	0.06	1.14	0.98	0.28	0.61
ALF-	0.74	0.10	1.18	0.04	1.13	1.14	0.36	0.67
SEM	0.047	0.065	0.243	0.018	0.059	0.084	0.111	0.068
Age								
10 WOA	1.84^a^	0.17^a^	1.21	0.00^b^	1.11^b^	0.86^b^	0.39	0.80^a^
15 WOA	0.28^b^	0.10^ab^	1.27	0.01^b^	0.87^c^	1.43^a^	0.29	0.60^b^
20 WOA	0.08^c^	0.03^b^	0.77	0.13^a^	1.43^a^	0.90^b^	0.27	0.52^b^
SEM	0.060	0.055	0.185	0.018	0.071	0.070	0.096	0.047
*P*-value								
Water access time	0.808	1.000	0.093	0.146	0.517	0.064	0.470	0.025
Access to alfalfa	0.314	0.733	0.576	0.132	0.993	0.241	0.617	0.506
Age	< 0.001	0.006	0.236	< 0.001	< 0.001	< 0.001	0.466	< 0.001
Water × alfalfa	0.688	0.964	0.674	0.194	0.467	0.913	0.677	0.937
Water × age	0.851	0.044	0.390	0.003	0.996	0.701	0.209	0.303
Alfalfa × age	0.901	0.850	0.674	0.137	0.511	0.638	0.576	0.936
Water × alfalfa × age	0.833	0.011	0.874	0.303	0.474	0.916	0.851	0.640

Abbreviation: WOA, weeks of age.

a–c Means within a column with no common superscript differences (*P* ≤ 0.05).

1 Feather cover score ranges from 0 (intact feathers) to 5 (completely denuded area).

2 3HR = 3 h water access between 07:30 am and 10:30 am h, 5HR = 5 h water access in 2 periods between 07:30 am and 11:00 am h and between 14:00 pm and 15:30 pm h, 8HR = 8 h water access during the entire light period. ALF+ = unlimited access to alfalfa straw, ALF- = no access to alfalfa straw. WOA = week of age.

The average feather cover contamination score was higher (indicating more dirtiness) in the 5HR and 8HR pullets than the 3HR pullets (0.94 and 0.95 vs. 0.57; *P* = 0.005; Table 8). The differences in feather cover contamination were caused primarily by differences in contamination of the neck, breast, belly, tail, and thighs. Similarly, the footpad contamination score was also higher (indication more dirtiness) in the 5HR and 8HR pullets than the 3HR pullets (0.97 and 0.83 vs. 0.05; *P* < 0.001). This indicates that both feather cover and foot pads were dirtier in the 5HR and 8HR pullets than the 3HR pullets. There were no differences in feather cover quality, feather cover or footpad contamination between the ALF+ and ALF- pullets.

**Table 8:** Effects of water access time, unlimited access to alfalfa straw, and age on contamination of feather cover and foot pads score.^1^

Treatment^2^	Neck	Breast	Belly	Back	Wings	Tail	Thighs	Average	Foot pads
Water access time									
3HR	0.27	0.64^b^	0.67^b^	0.77	0.81	0.52^b^	0.31	0.57^b^	0.05^b^
5HR	0.47	1.29^a^	1.57^a^	0.84	0.96	0.88^a^	0.60	0.94^a^	0.97^a^
8HR	0.49	1.28^a^	1.53^a^	0.85	0.94	0.93^a^	0.61	0.95^a^	0.83^a^
SEM	0.069	0.140	0.161	0.110	0.071	0.099	0.099	0.091	0.164
Access to alfalfa									
ALF+	0.42	1.12	1.26	0.83	0.94	0.77	0.54	0.84	0.62
ALF-	0.41	1.03	1.25	0.81	0.87	0.78	0.47	0.80	0.61
SEM	0.056	0.115	0.131	0.090	0.058	0.081	0.081	0.074	0.134
Age									
10 WOA	0.16^b^	0.67^c^	1.28^b^	0.38^b^	0.44^b^	0.58^b^	0.18	0.52^c^	0.26^c^
15 WOA	0.80^a^	1.48^a^	1.80^a^	1.17^a^	1.21^a^	1.19^a^	0.77	1.20^a^	1.01^a^
20 WOA	0.27^b^	1.07^b^	0.68^c^	0.92^a^	1.06^a^	0.54^b^	0.58	0.73^b^	0.54^b^
SEM	0.069	0.079	0.084	0.090	0.087	0.076	0.101	0.063	0.085
*P*-value									
Water access time	0.179	0.005	< 0.001	0.953	0.899	0.016	0.284	0.005	< 0.001
Access to alfalfa	0.518	0.454	0.688	0.884	0.993	0.891	0.526	0.844	0.587
Age	< 0.001	< 0.001	< 0.001	< 0.001	< 0.001	< 0.001	0.223	< 0.001	< 0.001
Water × alfalfa	0.800	0.809	0.937	0.301	0.999	0.661	0.817	0.743	0.564
Water × age	0.586	0.758	0.076	0.172	0.176	0.016	0.497	0.033	0.385
Alfalfa × age	0.196	0.995	0.124	0.964	0.135	0.987	0.641	0.750	0.236
Water × alfalfa × age	0.997	0.318	0.494	0.582	0.977	0.719	0.915	0.672	0.912

Abbreviation: WOA, weeks of age.

a–c Means within a column with no common superscript differences (*P* ≤ 0.05).

1 Contamination score ranges from 0 (clean) to 3 (filthy).

2 3HR = 3 h water access between 07:30 am and 10:30 am h, 5HR = 5 h water access in 2 periods between 07:30 am and 11:00 am h and between 14:00 pm and 15:30 pm h, 8HR = 8 h water access during the entire light period. ALF+ = unlimited access to alfalfa straw, ALF- = no access to alfalfa straw. WOA = week of age.

Over the rearing period, feather cover improved, with an average score of 0.80 at 10 WOA decreasing to an average score of 0.60 and 0.52 at 15 and 20 WOA (*P* < 0.001), respectively. Between 10 and 15 WOA, both the average feather cover and footpad contamination scores increased considerably, followed by a subsequent decrease between 15 and 20 WOA (*P* < 0.001).

### Behavioral Observations

The 5HR and 8HR pullets showed a higher rate of pecking at alfalfa straw (1.6% and 1.5% vs. 0.8%; *P* = 0.030) and more pecking at the drinking nipples (10.4% and 11.7% vs. 7.6%; *P* = 0.002) compared to the 3HR pullets (Table 9). Additionally, the 5HR and 8HR pullets showed less foraging (18.0% and 17.9% vs. 23.1%; *P* < 0.001) and less sitting on the perch (2.4% and 2.2% vs. 4.1%; *P* = 0.010) than the 3HR pullets.

**Table 9:** Effects of water access time, unlimited access to alfalfa straw, and age on behavior.^1^

Treatment^2^	Eat	Alfalfa	Drink	Stand	Sit	Walk	Forag	Comf	Dustb	Object	Bird	Sit perch	Stand perch
Water access time													
3HR	3.4	0.8^b^	7.6^b^	21.2	0.6	7.2	23.1^a^	3.2	0.5	23.2	0.2	4.1^a^	4.7
5HR	3.9	1.6^a^	10.4^a^	24.6	0.4	7.9	18.0^b^	3.1	0.4	23.2	0.2	2.4^b^	3.7
8HR	3.8	1.5^a^	11.7^a^	24.5	0.2	7.2	17.9^b^	2.7	0.2	24.1	0.1	2.2^b^	3.7
SEM	0.22	0.17	0.77	1.62	0.13	0.57	0.82	0.35	0.08	1.24	0.05	0.47	0.47
Access to alfalfa													
ALF+	3.6	2.5^a^	10.4	24.0	0.4	7.0	19.0	3.2	0.5	22.1	0.2	3.1	3.6
ALF-	3.7	0.0^b^	9.4	22.8	0.4	7.9	20.2	2.9	0.3	24.9	0.2	2.7	4.5
SEM	0.18	0.14	0.63	1.32	0.10	0.47	0.67	0.29	0.06	1.01	0.04	0.39	0.39
Age													
10 WOA	3.3	1.5^b^	7.3^b^	25.1	0.4	12.5^a^	18.2^b^	2.4b	0.5	22.1^b^	0.1	2.7	3.9^b^
15 WOA	3.5	1.8^a^	11.5^a^	23.5	0.4	6.1^b^	17.3^b^	2.9^b^	0.2	25.9^a^	0.2	3.1	3.4^b^
20 WOA	4.2	0.5^c^	10.9^a^	22.0	0.5	3.7^b^	23.5^a^	3.7^a^	0.4	22.6^b^	0.3	3.0	4.9^a^
SEM	0.53	0.14	0.51	0.99	0.10	0.47	0.84	0.24	0.09	0.84	0.06	0.23	0.22
*P*-value													
Water access time	0.773	0.030	0.002	0.209	0.254	0.827	< 0.001	0.630	0.179	0.834	0.450	0.010	0.219
Access to alfalfa	0.854	< 0.001	0.298	0.426	0.679	0.112	0.352	0.444	0.197	0.065	0.322	0.378	0.142
Age	0.428	< 0.001	< 0.001	0.069	0.533	< 0.001	< 0.001	0.001	0.148	0.002	0.302	0.420	< 0.001
Water × alfalfa	0.940	0.916	0.957	0.477	0.401	0.961	0.362	0.192	0.924	0.442	0.110	0.407	0.053
Water × age	0.972	0.101	0.208	0.422	0.658	0.780	0.250	0.217	0.525	0.947	0.177	0.015	0.768
Alfalfa × age	0.916	0.699	0.771	0.961	0.186	0.871	0.362	0.041	0.861	0.626	0.594	0.730	0.321
Water × alfalfa × age	0.982	1.000	0.892	0.299	0.483	0.495	0.945	0.168	0.781	0.393	0.455	0.236	0.502

Abbreviation: WOA, weeks of age.

a–c Means within a column with no common superscript differences (*P* ≤ 0.05).

1 Eat = pecking at feed at the feeding troughs; alfalfa = pecking at alfalfa in the bucket; drink = pecking at water at the drinking-nipples; stand = standing; sit = sitting; walk = walking; forag = foraging; comf = comfort; dustb = dust-bathing; object = stereotypical object-pecking; bird = pecking at other birds; sit perch = sitting on perch; stand perch = standing on perch.

2 3HR = 3 h water access between 07:30 am and 10:30 am h, 5HR = 5 h water access in 2 periods between 07:30 am and 11:00 am h and between 14:00 pm and 15:30 pm h, 8HR = 8 h water access during the entire light period. ALF+ = unlimited access to alfalfa straw, ALF- = no access to alfalfa straw. WOA = week of age.

The ALF+ pullets showed a tendency toward less object-pecking behavior (22.1% vs. 24.9%; *P* = 0.065) compared to the ALF- pullets. Furthermore, no differences in behavior were observed between the pullets with or without unlimited access to alfalfa straw.

As the pullets aged, there was an increase in pecking at the alfalfa straw at 15 WOA, followed by a decrease by 20 WOA. Additionally, with aging, the pullets spent more time on drinking, foraging, comfort, and standing on the perch. By contrast, with aging, the time spent on walking decreased, and standing tended to decreased. Object-pecking initially increased between 10 and 15 WOA, before decreasing again at 20 WOA.

## DISCUSSION

### Effect of Water Access Time

Pullets with 5 and 8 h of water access had higher water use, leading to an increased water-to-feed ratio. Notably, the water use and water-to-feed ratio in pullets with 5 h of water access in 2 periods did not differ from the pullets with consecutive 8 h access to water. This finding was unexpected, as it was anticipated that pullets with 5 h of water access would consume more water than the pullets with 3 h of water access but less than the pullets with 8 h of water access. The unexpected result can be explained by the measurements of water use throughout the day. These observations showed that pullets with access to 2 periods of water (5 h total) used more water during the second period (between 14:00 pm and 15:30 pm h). This observation suggests a potential compensation in water use during the second period, possibly in response to the preceding 3-h period without access to water, which could lead to impaired welfare.

Furthermore, it was observed that pullets with 3 h access to water had higher water consumption between 07:30 am and 09:30 am h than did pullets with 5 and 8 h access to water. This rapid drinking during a short period of water access was previously observed in an on-farm study (van Emous, 2022). Breeder pullets with a short period of water access (3 h) are conditioned to consume sufficient water quickly because they have experienced that they will then be deprived of water for a long period of time (21 h).

The average dry-matter content of the litter below the drinking-nipple line was 1.5 times lower in the pens housing pullets with 5 and 8 h of water access compared to the pens with 3 h of water access. This agrees with previous research of Hocking et al. (1993), who also found wet litter when pullets had unlimited access to water. The friability and wetness score of the litter was higher, indicating more caking and visual wetness of the litter, in the pens with 5 and 8 h of water access than those with 3 h of water access. This difference was likely directly caused by the higher water use, specifically by water spillage in the litter. Litter condition plays a critical role in leg health and welfare, as poorer litter quality can contribute to the occurrence of hock burns and food pad dermatitis (Kaukonen et al., 2016).

It was surprising that no differences were observed in the dry-matter content of the fresh feces among the different water access time. The initial expectation was that higher water use would lead to increased water consumption, in turn resulting in wetter fresh feces, as found in broilers by van Harn et al. (2019). The literature indicates that controlled feed amounts for breeder pullets may lead to abnormal behaviors, such as higher water intake or playing with water and thus spilling it (Hocking et al., 1996, 2001; Savory and Kostal, 1996; de Jong et al., 2002). In the current study, despite the lack of differences in the dry-matter content of the fresh feces, the dry-matter content below the drinking-nipple line was much lower in pens with 5 and 8 h of water access. It is therefore concluded that most of the higher water use did not result in physical water consumption but rather led to spillage in the litter.

The dry-matter content of the litter decreased between 7 and 17 WOA but increased again at 20 WOA due to the addition of fresh wood shavings to pens with poor litter quality. In the pens with 5 and 8 h of water access, an average of 1.5 and 2.3 times fresh litter, respectively (2 kg per addition), was introduced to enhance litter quality to support animal welfare. Additionally, outdoor weather conditions, characterized by dry spring weather, positively affected the litter quality between 17 and 20 WOA. This pattern in dry-matter content of the litter was also reflected in the visual assessment of the litter quality. The friability and wetness scores were clearly higher (indicating lower litter quality) in the pens with 5 and 8 h of water access. Wetter litter tends to be darker and results in caking of the top layer, making it visually wetter and less loose, according to previous research with broilers by van Harn et al. (2019).

The dry-matter content of the fresh feces increased during the experimental period from 21.5% to almost 24.5%. This shift was due to the higher feed allowance (increasing from 53 to 85 g between 7 and 20 WOA) and a lower water-to-feed ratio (from 2.6 to 2.2 between 7 and 20 WOA). The dry-matter content of the fresh feces in the current experiment is consistent with prior research on laying hens, where 23% was reported (van Middelkoop 1993). In comparison, broilers tend to have a somewhat higher DM content (28%) in fresh feces due to the higher feed intake and lower water-to-feed ratio (Aarnink et al., 2016).

Average feather cover, or more specific on the belly and tail, was poorer in the pullets with 5 and 8 h of water access compared to the pullets with 3 h of water access. The explanation for the poorer feather cover on the belly is that pullets in the pens with 5 and 8 h of water access were in contact with poor litter (i.e., wet litter) for longer time periods. Pullets in these pens, exposed to worse litter conditions, spent part of the day, and often the entire night, en masse on the wet litter and less on the perches. This prolonged contact with the poor litter likely caused the feathers to become moist and dirty, and it is postulated that feathers were pulled out of the skin due to adherence to the wet litter. The poorer feather cover on the tail can be explained by pulling feathers out as result of feather licking.

The average contamination of feather cover and foot pads was clearly worse in the pullets with 5 and 8 h of water access compared to the pullets with 3 h of water access. This was primarily caused by direct contact between the breast, belly, and foot pads with the wet litter. Additionally, the tail was also dirtier, possibly due to contamination during feeding time because of crowding behavior around the feeding troughs.

Unexpectedly, there were no differences in feed allowance between the different water access time. In practice, it is often indicated that higher water consumption requires a higher feed allowance to achieve the same BW growth. It is likely that when pullets drink more, the feed is diluted, causing the nutrients to be absorbed less efficiently, as the feed “flushes” with a negative effect on body growth (Nielsen et al., 2011). In the current study, this was not the case, indicating that the pullets may not have physically absorbed the excess of water and did not experience the flushing effect.

In the intermediate and experimental periods, alfalfa use in the pens with 5 and 8 h of water access was on average 1.6 and 2.4 times higher, respectively, than the alfalfa use in pens with 3 h of water access. Most of the alfalfa straw appeared not to be spilled (van Emous, 2023, personal observation), but it cannot be ruled out completely that this occurred more in the pens with 5 and 8 h of water access. In pens with poor litter and almost completely caked litter (only in the pens with 5 and 8 h of water access), not enough loose litter was available as foraging material. It is hypothesized that pullets in pens with poor litter use the alfalfa straw as foraging material, resulting in higher use of it. This was confirmed by the behavioral observations, where the pullets in pens with 5 and 8 h of water access showed more pecking behavior at the alfalfa straw buckets.

There were large differences in alfalfa straw use between pens within the same treatment (data not shown). For example, within the pens with 8 h access to water, daily alfalfa use varied between 2.8 and 5.9 g per pullet. This large difference in alfalfa straw use may be related to the behavioral synchronization of the pullets. We know that several behaviors (e.g., foraging, dust-bathing, and eating) are often performed synchronously (Appleby et al., 2004), resulting in more alfalfa straw pecking in specific pens.

The pullets that were provided with water for 5 or 8 h per day showed more drinking behavior. The fact that the pullets spent more time on drinking in the pens with 5 or 8 h access to water was also evident from the higher water use and lower dry-matter content of the litter. The pullets that had water available for a longer period were more focused on water-related activities, leading to increased spillage and subsequently poorer litter. This is consistent with previous research by Hocking (1993), who also observed poorer litter in pullets with unrestricted access to water.

Due to the poorer litter, the pullets with 5 and 8 h access to water showed less foraging behavior. This is in accordance with research by Riber et al. (2021), who also found in an experiment with different fiber-rich raw materials less foraging in one of the treatments, with poorer litter. It is notable that the pullets with 5 and 8 h of water access spent less time on the perch. The expectation was that, due to the poorer litter quality, that pullets would sit more on the perches to avoid the poor litter. It is hypothesized that the pullets were colder because of the wetter litter, which caused them to sit close together on the litter to find warmth.

### Effect of Unlimited Access to Alfalfa Straw

Providing unlimited alfalfa straw did not affect the DM content of the litter, DM content of the fresh feces, litter friability score, or litter wetness score. Due to the lack of comparable research with feeding alfalfa straw to poultry in relation to litter quality, there were no expectations for this trait. It can be reasoned that providing alfalfa straw can have a positive effect on litter quality because the animals also spill the alfalfa straw on the litter, which in turn increases foraging behavior in the litter. Litter quality, however, was throughout most of the experimental period very poor in the pens with 5 and 8 h access to water, and the pullets were hindered from performing foraging behavior.

There were no differences in feather cover quality between pullets receiving unlimited alfalfa straw and those without. This phenomenon can be attributed to the absence of differences in litter quality (DM content and visual scores) in pens where alfalfa straw was either present or absent. 

It was unexpected that throughout nearly the entire rearing phase, no differences in feed allowance were found between the pullets with and without unlimited access to alfalfa straw. During the last 2 wk of the rearing period, a small reduction in feed allowance was found for pullets with unlimited access to alfalfa straw. The initial expectation was that additional crude protein (17.5%; CVB, 2018) provided through alfalfa straw would result in a decreased need for feed to achieve the same BW pullet profile. It is hypothesized that the pullets primarily consumed the alfalfa stems, and that most of the alfalfa leaves, containing the highest energy and protein levels, may have been lost. Furthermore, it is plausible that the pullets, due to the access to alfalfa straw, showed increasing activity, such as pecking at the alfalfa straw, which could have resulted in higher energy requirements.

The BW uniformity in pullets with unlimited access to alfalfa straw was slightly worse compared to pullets without access to alfalfa straw. This could be attributed to individual pullets within pens with a higher preference for alfalfa, leading them to ingest more alfalfa and potentially grow faster. Furthermore, the BW uniformity of the animals deteriorated throughout the rearing phase due to the absence of grading in the pullets. Under commercial circumstances, grading is typically conducted at different ages during the rearing period, where smaller pullets are separated in a special pen and provided with additional feed. The breeding company recommends grading when the CV exceeds 10% (Aviagen, 2021).

The pullets with unlimited access to alfalfa showed a tendency to reduced object-pecking, suggesting an improvement in their overall behavior and welfare. There is no comparable research on the effects of unlimited access to alfalfa on animal behavior in broiler breeders. In laying hens, it is established that providing unlimited alfalfa as enrichment material contributes to preventing feather pecking, a form of abnormal pecking behavior, thereby resulting in improved feather cover (Schreiter et al., 2020; Tainika and Şekeroğlu, 2021).

## CONCLUSIONS

The experiment demonstrated that providing broiler breeder pullets with extended water access, via nipple drinkers with drip cups, results in increased water use, poor litter quality, poor feather cover, and increased contamination of both feather cover and foot pads. The lower DM content observed in the litter beneath the drinkers, along with the similar DM content in fresh feces, among pullets with extended water access time, suggest that water spillage is more likely than physical increased water intake. Additionally, the study indicated that providing alfalfa resulted in a slightly reduced feed allowance at the end of the rearing period, slightly poorer BW uniformity, and decreased object-pecking behavior.
